# Medical and Physical Intelligence

**Published:** 1822-12

**Authors:** 


### MEDICAL AND PHYSICAL INTELLIGENCE.

1. *Some Account of the Medical Schools, &c. at Berlin.—(Extract of a Letter to* Dr. GRANVILLE, *from a Physician at Berlin.)*

* * * * IN the first place, permit me to observe that Prussia, like the tyrannical empire of Bonaparte, of which it has preserved many of the maxims and manners, presents the curious spectacle of an absolute, despotic, military government, which protects the sciences and literary men, and which represses, at the same time, by force, those disorders which its liberality and condescension towards the literati might otherwise produce. Thus, in Berlin are barracks containing from 24 to 25,000 soldiers, in a city of 180,000 souls, which are destined to restrain by force of arms the expression of public opinion; and, at the same time that some dozens of scholars are shut up in prison, the government, you will observe, sends into England, at its own expense, young professors, (one of whom is on his way thither,) and gives reception to foreign literati, and also to fugitives, (like Mr. Accum, who was named the other day professor of chemistry, in an inferior grade of the Polytechnic School.)

Here the professors of the University can choose the particular branch they wish to teach, and, as all the lectures and exercises are paid, there is a certain emulation to obtain them. To see the catalogue of lectures which are given in the University, you would believe that there were more professors than scholars; and, indeed, there are very many of the former, but all the lectures announced in the catalogue do not take place, because a sufficient number of auditors is very often wanting, and without which the lecture is not given. It is also to be observed, that professors, either extraordinary or adjunct, or particular, go also to the University to teach, and these are paid less; but they cannot avail themselves of the means of the University to give demonstrations. Comparative anatomy, according to my opinion, is that which is best taught in this University, by the celebrated RUDOLPHI, whose scholars are twice as numerous as those of BLAINVILLE, although the proportion is less than half of that of the scholars of Paris, and that there are no strangers here. Rudolphi has enriched the Walterian museum with a precious collection of preparations in comparative anatomy, and, by the celebrity of his name, attracts from all parts of the world curiosities, with which he every day enriches the museum. He also gives lessons of philosophy and nosology. He is familiar with the sciences of all nations, and possesses a medical library, excellently furnished with respect to anatomy and physiology, both human and comparative. He appreciates the Italian naturalists more than the English; and the only defect I can find in him is this prejudice against the English and England, which country he does not desire to see, although he has been, and intends soon to return, to Paris. Learned, profound, and jovial, he unites to a sound doctrine great affability, which renders him a very different man from what you would suppose him to be, looking at the portrait which is given of him in a recent Number of the "Journal Complementaire," and which, as he himself says, is really a caricature. He has been my familiar friend, as it were, with whom I have conversed much; the others, through want of politeness, not having learnt to get rid of their national haughtiness, and being only accustomed to converse with their scholars, with whom they always preserve a certain distance. The collection of intestinal worms of all animals belonging to Rudolphi is greatly superior to every other, and in truth is admirable; for my own part, I was contented with being shown the more rare kinds found in the human body. But, besides those of his own collecting, he receives continual additions, from foreign parts, as well as from his friends

The Cabinet of Natural History is one of the richest, although its existence is but of a recent date. In order to increase the contents, there is an ordinance in Prussia for the proprietors of ambulating menageries to sell to the royal cabinets all the animals that die. There are two young men paid also to prepare skeletons. M. LICHENSTEIN, director of the Museum of Natural History, has collected a great deal, but the chief part has been obtained from private collections. It is to be observed that, on the days when the museum is open, the masters and mistresses, with their respective scholars, may go to receive the benefit.

[To be continued.]

---

### 2. *On the Use of Muriate of Soda in making Anatomical Preparations. (Letter from* Mr. COOKE.*)*

Mr. SHAW, in his valuable work entitled "A Manual of Anatomy," states that he would never put up valuable preparations in solutions of corrosive sublimate, of alum, or of muriate of soda; "because," says he, "a change always takes place in the appearance of the preparation in the course of a few months."* I have had no experience as to the efficacy of any of these solutions, except that of muriate of soda; but, in reference to this fluid, the gentleman in question certainly has made an unguarded assertion. In the Medical and Physical Journal for March 1817, I published an account of experiments on the preservation of anatomical specimens in brine; and a further account is given in the Transactions of the Society of Arts for 1819, in consequence of that Society having voted me their silver medal. Permit me now, when more than six years have elapsed, to affirm that no change whatever has taken place in any one of the specimens then put up. The fluid is perfectly transparent, and the appearances are precisely as they were described in the publications alluded to. I think it right to mention that six or eight of the preparations remained at the rooms of the Society of Arts for, I think, nearly twelve months, before the sanction of that institution was afforded. Since the year 1816, it has not been convenient for me to enlarge my collection much, and I have put up only a few specimens with care; but I have had a very considerable number of specimens in bottles and jars, only waiting a favourable op-

* Second Edition, p. 431.

portunity of bringing them forward. I do not intend to maintain that we are yet capable of preserving all kinds of texture in the saline solution, nor am I at this moment prepared to extend the inquiry; but I think it due to myself, and to the profession, to state, that all the specimens, of which I have laid an account before the public, are still as perfect as possible, and not the least perceptible evaporation has taken place. Indeed, when I detailed the particulars in your Journal, the fluid in some of the specimens was a little turbid, but it afterwards became perfectly bright, and remains so. I am not conscious of having any thing to retract from the papers alluded to. It is proper to remind your readers, however, that in the first instance I employed a purer kind of muriate of soda than that of commerce, and I am not prepared to affirm that the latter is equally efficacious.

Though Mr. Shaw's work has arrived at a second edition, it did not fall into my hands till this morning; but, as I think it of importance to correct the statement he has made, you will oblige me by inserting this letter in your next Number. Any professional gentleman is perfectly welcome to see the specimens.

*67, Great Prescot-street; Oct.* 12, 1822.

---

### 3. *Note from* Mr. SHAW, *on the same Subject.*

Mr. COOKE, of Prescot-street, was so kind, a few days ago, as to show me some preparations which he has preserved in a solution of common salt since 1816. They are still in a state of excellent preservation, and little or no change appears to have taken place in them, excepting a slight action on the animal matter, similar to what happens when it is exposed to a weak solution of muriatic acid. Though prejudice in favour of the method I have long pursued in putting up preparations will probably induce me to continue the same plan, yet I must do Mr. Cooke the justice to say, that there may be a question whether the change produced upon animal matter by the action of the salt is more disadvantageous in a preparation intended to exhibit morbid structure, than that caused by spirits.

*October* 16.

---

### 4. *Bleeding proposed as a Remedy for Poisoning by Prussic Acid. (Extract of a Letter from* Mr. HUME, *of Long-Acre.)*

In a letter which I lately received from a medical friend in Cambridgeshire, who had requested me to ascertain by an analysis that a valuable horse had been destroyed by arsenic, which question naturally led to the subject of *poisons*, he mentions a case in which the *hydrocyanic acid* was given to a dog. As the account may be interesting to the readers of your valuable pages, I shall give it in his own words, without any comment.

" Wanting to destroy a greyhound, which was given to me, for the sake of my apprentice, to inspect the viscera, I thought the quickest mode of depriving him of life was to give him half a drachm of hydrocyanic acid, which I did by mixing it in a little water, and pouring it into his throat. He got about half of it, and in a few seconds a convulsive action of the diaphragm came on, producing a husky cough: he trembled, lost the use of his hind legs, and fell backward much convulsed, and I expected he was quite dead. A second convulsive fit came on, and he was lingering in great agony, which induced me to open the jugular vein, in the expectation it would soon dispatch him. He bled profusely, and, to my utter astonishment, he rose up, shook himself, stood firm, and seemed quite well! I have no doubt he would have entirely recovered, had I allowed him; but, unwilling to put him, as it were, to the agony of a second death, I gave another dose of the prussic acid, when he gave a most hideous yell, leaped up very high, fell backwards, and died.

"The knowledge of this circumstance in your hands, perhaps may lead you, or some of your numerous scientific friends, to prosecute the inquiry, to know if *poisoning by prussic acid* may not be successfully prevented by *copious bleeding*."

*To the Editors of the*
*London Medical and Physical Journal.*

---

5. *On removing Opium from the Stomach with a Syringe.*
*(Extract of a Letter from* Mr. BUSH, *of Frome.)*

When I published, in your Journal, my paper on withdrawing Opium from the Stomach by a Tube fixed to a common Syringe, I had never read nor heard of Mr. JUKES's apparatus for the same purpose. The melancholy case that gave rise to my invention (if it deserves the name,) occurred on the 13th of July; and on the evening of the same day I had formed the apparatus I afterwards gave a description of to the public. Though Mr. Jukes may have published a few days before my instrument was constructed, an account of it had never reached the medical men of this place; for, though the patient was under the immediate care of the gentleman named in my paper, all the professional men in the town were acquainted with it, and not one word of Mr. Jukes, or his apparatus, passed between us:—so that, if the word "piracy," in Mr. Jukes's paper, is pointed at me, I must repel it, and sincerely assure that gentleman it does not apply.

Mr. Jukes thanks me for suggesting the best position to place the patient in to effect the removal of the poison, and he says he now uses the syringe instead of the bottle,—I am glad he approves of my plan. On the size of the syringe, I would say one word by way of question: —Would it be quite prudent to inject into the stomach a quart of water, under the circumstance of opium having been swallowed to a dangerous extent? Would there not be some danger, especially if the stomach was previously full, of forcing the poison beyond the reach of the exhauster, and occasioning the very effect intended to be prevented? I humbly submit, that the introduction of a small quantity of water (only half a pint, or a little more at furthest,) would be a safer, and consequently a more adviseable plan.

6. *Remarks on Inflammation in the Chest. (Extract of a Letter from* JOSEPH MORLEY, Esq. Surgeon.)

As peripneumonia frequently takes place at this season of the year, and a great number of cases having fallen to my lot during my residence at Wellingore, in the county of Lincoln, I have at length discovered a characteristic symptom, which will enable practitioners in medicine to discover when the inflammatory stage has ceased, and the purulent commenced. The discovery I consider of the greatest importance, because it will stay (or at least moderate) the further necessity of bleeding, the remedy at present principally depended upon in the inflammatory stage, and, if continued when an abscess has formed, frequently proves fatal. It will also enable the medical attendant to give peremptory orders for the patient to be kept in bed, with the chest well elevated; for, if this is neglected, immediate death sometimes takes place from suffocation.

The change from the inflammatory to the purulent stage may be ascertained to a certainty by the following test:—Place a hand upon each side of the chest, at the same time order the patient to articulate: if the inflammatory stage is still going on, a peculiar jar or vibration will be felt by the hands; if an abscess has formed, no such sensation will be experienced. If only one side is affected, the motion will be lost on that side, and distinctly felt on the other. In empyema, the vibration is lost also. The peculiar sensation to which I allude may be distinctly perceived by any person in health placing a hand on each side of the chest, at the same time coughing or speaking. In the female it is not so distinct as in the male, though very perceptible in both.

### METEOROLOGICAL JOURNAL.

By Messrs. William Harris and Co. 50, Holborn, London.

*From October 20, 1822, to November 19, 1822.*

| | MOON. | Rain gauge | THERM. | | | BAROM. | | DE LUC'S HYG. | | WIND. | | ATMOSPHERIC VARIATION. | | |
|---|---|---|---|---|---|---|---|---|---|---|---|---|---|---|
| | | | 9 A.M. | MAX. | MIN. | 9 A.M. | 10P.M. | 9 A.M. | 10P.M. | 9 A.M. | 10P.M. | 9 A.M. | 2 P.M. | 10P.M. |
| Oct. | | | | | | | | | | | | | | |
| 20 | | .42 | 55 | 58 | 48 | 29.40 | 29.28 | 77 | 76 | W | WSW | Fine | Rain | |
| 21 | | .04 | 52 | 56 | 40 | 29.37 | 29.41 | 73 | 73 | WSW | WSW | Fine | Rain | Cloud. |
| 22 | | | 46 | 46 | 41 | 29.57 | 29.68 | 76 | 71 | W | W | Fine | | |
| 23 | ☾ | | 47 | 56 | 53 | 29.52 | 29.35 | 81 | 70 | E | SE | Fine | | Cloud. |
| 24 | | .10 | 55 | 62 | 53 | 29.23 | 29.21 | 69 | 76 | S | S | Fine | Cloud. | Rain |
| 25 | | | 55 | 63 | 49 | 29.30 | 29.45 | 80 | 75 | SW | SSW | Fine | | |
| 26 | | | 51 | 59 | 48 | 29.38 | 29.28 | 80 | 68 | SSE | SSE | Fine | | |
| 27 | | | 51 | 57 | 39 | 29.45 | 29.50 | 72 | 75 | WNW | W | Fine | Fine | Foggy |
| 28 | | | 43 | 49 | 46 | 29.62 | 29.75 | 79 | 70 | SE | SW | Fine | | |
| 29 | | .22 | 54 | 56 | 42 | 29.73 | 29.84 | 85 | 75 | SW | NE | Rain | Cloud. | Fog |
| 30 | ○ | | 55 | 59 | 50 | 29.83 | 29.64 | 87 | 84 | SE | SSW | Fog | Cloud. | Cloud. |
| 31 | | | 53 | 60 | 52 | 29.53 | 29.60 | 78 | 76 | S | SW | Fine | | |
| Nov | | | | | | | | | | | | | | |
| 1 | | .03 | 54 | 64 | 57 | 29.70 | 29.68 | 75 | 68 | SW | SW | Fine | Cloud. | Rain |
| 2 | | | 60 | 65 | 52 | 29.52 | 29.68 | 64 | 68 | SW | WSW | Fine | | |
| 3 | | | 56 | 58 | 42 | 29.81 | 30.00 | 72 | 69 | W | W | Fine | Cloud. | Fine |
| 4 | | | 48 | 51 | 46 | 30.10 | 30 09 | 78 | 65 | W | W | Fine | | |
| 5 | | | 50 | 54 | 42 | 30.06 | 30.03 | 72 | 67 | W | WSW | Fine | | |
| 6 | ☽ | | 52 | 57 | 46 | 30 00 | 29.92 | 72 | 65 | W | W | Fine | | |
| 7 | | | 58 | 64 | 42 | 29.78 | 29.71 | 72 | 66 | WSW | WNW | Fine | | |
| 8 | | .30 | 45 | 55 | 36 | 29.74 | 29.81 | 75 | 67 | N | NE | Rain | | |
| 9 | | .04 | 38 | 42 | 46 | 29.81 | 29.67 | 76 | 79 | NE | SSE | Fog | Cloud. | Rain |
| 10 | | .03 | 52 | 54 | 42 | 29.65 | 29.95 | 91 | 77 | NE | NE | Fine | Rain | Cloud. |
| 11 | | | 43 | 52 | 48 | 30.07 | 30.06 | 85 | 72 | NE | SW | Fine | Fine | |
| 12 | | | 52 | 52 | 45 | 29.92 | 29.76 | 77 | 83 | SW | SW | Fine | | Cloud. |
| 13 | ● | | 48 | 52 | 43 | 29.73 | 29.62 | 76 | 75 | N | NW | Overcast | Fine | |
| 14 | | .24 | 46 | 52 | 44 | 29.22 | 29.36 | 87 | 67 | WNW | WSW | Rain | | Sl. Fog |
| 15 | | .95 | 45 | 50 | 40 | 29.09 | 29.26 | 72 | 63 | WSW | W | Rain | Cloud. | Fine |
| 16 | | .26 | 42 | 42 | 34 | 29.10 | 29.29 | 96 | 83 | E | WNW | Rain | | Cloud. |
| 17 | | | 41 | 48 | 47 | 29.45 | 29.47 | 74 | 75 | WSW | SW | Fine | | |
| 18 | | .15 | 53 | 56 | 44 | 29.40 | 29.53 | 88 | 79 | WSW | SW | Rain | Cloud. | Fi. Ra. |
| 19 | | | 48 | 53 | 56 | 29.71 | 29.60 | 82 | 76 | SW | WSW | Cloudy | Cloud. | Cloud. |

The quantity of rain fallen in the month of October, was 3 inches and 93.100ths.

